# Supplementary material for: The Clonal Spread and Persistence of Campylobacter in Danish Broiler Farms and Its Association with Human Infections
Source: Pathogens. 2025 Aug 19;14(8):821. doi: 10.3390/pathogens14080821 (PMC12389240; doi:10.3390/pathogens14080821)
Supplement: Supplementary file 1 [file pathogens-14-00821-s001.zip › pathogens-3759695-supplementary.pdf]

Table S1: Cluster types across farms, houses and rotations

Farm	House		Rotation	Sequences		Types		
				Total	Per rotation		Total	Campylobacter (cgMLST) Type
**C1**	1	**2020**	5<br>6	**13**	9<br>4	3<br>2	**3**	ST42-1, ST50-1, ST855-1
	2		4<br>5<br>6	**17**	10<br>4<br>3	2<br>2<br>2	**4**	ST19-1, ST607-1<br>ST50-1<br>ST855-1
	3		4<br>6	**8**	**2**<br>**6**	1<br>1	**2**	ST1268-1<br>ST50-1
	4		5<br>6	**6**	3<br>3	1<br>1	**2**	ST855-1<br>ST50-1
	5		5<br>6	**7**	4<br>3	2<br>1	**2**	ST855-1<br>ST50-1
	6		6*	**3**	-	-	**1**	ST50-1
**C2**	1		5<br>6	**13**	10<br>3	1<br>1	**2**	ST45-1,<br>ST267-1
	2		5<br>6	**9**	6<br>3	2<br>1	**3**	ST45-1, ST829-1<br>ST48-1
	3		5<br>6	**11**	7<br>4	1<br>2	**3**	ST45-1;<br>ST854-1, ST22-1
	4		5<br>6	**13**	10<br>3	2<br>1	**3**	ST45-1, ST829-1<br>ST48-1
	5		5	**6**	-	-	**1**	ST45-1
	2	2021	5<br>6	**6**	3<br>3	1<br>2	**3**	ST45-15<br>ST21-10, ST45-8
	3		5<br>6<br>7	**10**	4<br>3<br>3	1<br>2<br>1	**4**	ST45-15<br>ST21-10, ST48-3<br>ST11341-1
**C3**	1	2020	4	**9**	-	-	**1**	ST9855-1
	2		5	**10**	-	-	**2**	ST10628-1, ST1583-1
	3		5<br>6	**11**	10<br>1	1<br>1	**2**	ST10628-1<br>ST50-5
	4		5<br>6	**12**	10<br>2	1<br>1	**2**	ST10628-1<br>ST50-5
**C4**	1		5<br>6	**18**	10<br>8	1<br>2	**3**	ST832-1<br>ST257-2, ST19-5
	2		5<br>7	**13**	10<br>3	1<br>1	**2**	ST257-1<br>ST19-5
	2	2021	5	**3**	-	-	**1**	ST11151-2
**C5**	1	2020	7	**3**	-	-	**2**	ST854-2, ST854-3
	2		5	2	-	-	**1**	ST2655-1
**C6**	1		6	3	-	-	**1**	ST441-1
	2		6	3	-	-	**1**	ST1701-1
**C7**	1		4<br>5<br>6<br>7	**23**	2<br>8<br>10<br>3	1<br>2<br>1<br>1	**3**	ST4800-1<br>ST50-2, ST230 -1<br>ST50-2<br>ST50-2

Farm	House		Rotation	Sequences		Types		
				Total	Per rotation		Total	Campylobacter (cgMLST) Type
	2		4<br>5<br>6	**20**	1<br>9<br>10	1<br>2<br>2	**5**	ST4800-1<br>ST21-1, ST1153-1<br>ST1153-2, ST50-2
	1	2021	4<br>6	**9**	4<br>5	1<br>1	**2**	ST583-2<br>ST890-1
	2		4<br>5<br>6	**10**	4<br>1<br>5	1<br>1<br>1	**2**	ST583-2<br>ST7355-1<br>ST7355-1
**F1**	1	2020	5	**6**			**2**	ST257-4, ST22-2
	2		3<br>5	**10**	1<br>7	1<br>2	**3**	ST19-2<br>ST1056-1, ST22-2
	3		4	**5**	-	-	**1**	ST257-4
	4		4	**1**	-	-	**1**	ST257-4
	1	2021	4	**3**	-	-	**2**	ST42-3, ST257-5
	2		3<br>4	**6**	3<br>3	1<br>1	**2**	ST61-1<br>ST257-5
**F2**	1	2020	3<br>4	**24**	6<br>18	2<br>3	**5**	ST267-3, ST22-3<br>ST1585-1, ST1947-1, ST45-2
	2		2<br>3	**20**	10<br>10	4<br>1	**5**	ST1585-1, ST1583-2, ST3276-1, C. lari ST58-1<br>ST22-3
	1	2021	4	**3**	-	-	**1**	ST267-3
	2		4<br>5	**6**	3<br>3	2<br>1	**3**	ST45-5, ST267-3<br>ST448-1
**F4**	1	2021	3<br>5	**6**	3<br>3	3<br>1	**4**	ST45-6, ST45-7, ST21-8<br>ST257-3
	2		3	**1**	-	-	**1**	ST6175-1
**F5**	1		4<br>5	**6**	4<br>2	2<br>2	**4**	ST48-4, ST1830-1<br>ST475-2, ST257-5
	2		4	**1**	-	-	**1**	ST48-4
**F6**	1		5<br>6<br>7	**10**	4<br>3<br>3	3<br>1<br>1	**4**	ST354-2, ST45-16, C. lari ST28-1<br>ST827-1<br>ST827-1
	2		5<br>6<br>7	**10**	3<br>4<br>3	1<br>3<br>1	**4**	ST607-2<br>ST607-2, ST8195-1, ST45-16<br>ST827-1
**F7**	1		5<br>6<br>7<br>8	**13**	3<br>3<br>3<br>4	1<br>3<br>2<br>3	**6**	ST45-14<br>ST45-14, ST45-9, ST45-10<br>ST2229-1, ST10025-1<br>ST10025-1, ST1445-2, ST2229-1
	2		6<br>7<br>8<br>9	**15**	3<br>4<br>4<br>4	1<br>2<br>3<br>3	**5**	ST45-14<br>ST2229-1, ST1445-1<br>ST1445-1, ST10025-2, ST2229-1<br>ST2229-1, ST1445-1, ST1595-2
**F8**	1		4<br>5	**7**	3<br>4	1<br>2	**2**	ST475-2<br>ST475-2, ST1595-2
**F9**	1		3<br>4<br>5	**9**	3<br>3<br>3	2<br>2<br>1	**4**	ST6175-1, ST475-2<br>ST45-13, ST267-4<br>ST267-4
	2		3<br>4<br>5<br>6	**13**	3<br>4<br>3<br>3	2<br>3<br>1<br>2	**5**	ST267-4, ST475-2<br>ST267-4, ST1595-2, ST45-13<br>ST267-4<br>ST267-4, ST6175-1
	3		3<br>4<br>5	**11**	3<br>4<br>4	1<br>3<br>4	**4**	ST6175-1<br>ST267-4, ST475-2, ST1595-2<br>ST1595-2, ST475-2, ST6175-1, ST267-4

Farm	House		Rotation	Sequences		Types		
				Total	Per rotation		Total	Campylobacter (cgMLST) Type
**F10**	1		3<br>4<br>5	**10**	3<br>3<br>4	1<br>1<br>2	**3**	ST475-2<br>ST45-12<br>ST475-2, ST1595-2
	2		4<br>5	**6**	3<br>3	1<br>1	**1**	ST45-12<br>ST45-12
**C8**	1	2020	6	**3**	-	-	**2**	ST42-4, ST19-6
	2		6	**3**	-	-	**2**	ST45-3, ST45-4
	3		6	**3**	-	-	**2**	ST42-4, ST19-6
	4		6	**4**	-	-	**3**	ST42-4, ST2507-1, ST21-2
	5		5<br>6	**4**	1<br>3	1<br>1	**2**	C. lari ST71-1<br>ST42-4
	1	2021	6	**4**	-	-	**1**	ST1701-2
	5		6<br>7	**9**	4<br>5	1<br>2	**2**	ST825-1<br>ST11319-1, ST825-1
**C9**	1	2020	5	**20**			**1**	ST21-9
	2		5<br>6<br>7	**24**	17<br>4<br>3	1<br>4<br>1	**6**	ST21-9<br>ST2274-1, ST7355-1, ST21-4, ST11-1<br>ST21-3
**C10**	1	2020	6<br>7	**6**	3<br>3	1<br>2	**3**	ST7355-1<br>ST583-1, ST2274-1
	2		5<br>6<br>7	**13**	2<br>8<br>3	1<br>2<br>1	**3**	ST7355-1<br>ST583-1, ST2274-1<br>ST2274-1
	3		5<br>6<br>7	**18**	6<br>6<br>6	1<br>3<br>2	**5**	ST7355-1<br>ST872-1, ST7355-1, ST10638-1<br>ST583-1, ST2274-1
	4		5<br>6<br>7	**21**	9<br>9<br>3	1<br>2<br>2	**4**	ST7355-1<br>ST583-1, ST7355-1<br>ST38-1, ST2274-1
	5		5<br>6<br>7	**21**	7<br>8<br>6	1<br>3<br>1	**4**	ST7355-1<br>ST583-1, ST872-1, ST2274-1<br>ST2274-1
	1	2021	5<br>7	**8**	4<br>4	1<br>1	**2**	ST50-4<br>ST11151-1
	2		4<br>5<br>6<br>7<br>8	**15**	3<br>1<br>5<br>3<br>3	1<br>1<br>2<br>1<br>1	**5**	ST19-4<br>ST257-6<br>ST52-1, ST1595-3<br>ST52-1<br>ST21-11
	3		5<br>7	**7**	4<br>3	1<br>1	**2**	ST7355-1<br>ST5-1
	4		5<br>6	**10**	5<br>5	2<br>2	**4**	ST50-4, ST1595-3<br>ST860-1**,** ST52-1
	5		5<br>6	**8**	4<br>4	3<br>2	**4**	ST52-1, ST45-17, ST1595-3<br>ST52-1, ST860-1
**C11**	1	2020	4<br>6<br>7	**19**	10<br>3<br>6	2<br>1<br>1	**4**	ST1080-1, C. lari ST21-1<br>ST48-2<br>ST475-1
	2		7	**3**	-	-	**1**	ST475-1
**C12**	1	2020	5<br>6<br>7	**17**	10<br>3<br>4	2<br>1<br>1	**4**	ST1595-1, ST354-1<br>ST22-2<br>ST230-2
	2		5<br>6	**13**	10<br>3	1<br>1	**2**	ST122-1<br>ST22-2
	3		5<br>6	**13**	10<br>3	1<br>1	**2**	ST122-1<br>ST22-2

Farm	House		Rotation	Sequences		Types		
				Total	Per rotation		Total	Campylobacter (cgMLST) Type
	4		4	**19**	5	1	**4**	ST42-2
			5		8	1		ST122-1
			6		3	1		ST22-2
			7		3	1		ST10042-1
	1	2021	5	**3**	-	-	**1**	ST11129-1
	4		4	**1**	-	-	**1**	ST137-2
**C13**	4	2020	5		1		**1**	ST21-9
**C14**	**1**	2020	5	**12**	3	1	**3**	ST137-1
			6		3	1		ST2254-1
			7		6	1		ST45-11
	**2**		6	**10**	3	1	**2**	ST267-2
			7		7	1		ST45-11
	**3**		6	**11**	5	2	**3**	ST2254-1, ST441-2
			7		6	1		ST45-11
	**4**		5	**28**	19	1	**4**	ST21-5
			6		3	1		ST2254-1
			7		6	2		ST2274-1, ST45-11
	**1**	2021	5	**4**	-	-	**2**	ST9291-1, ST21-7
	**4**		6	**8**	7	1	**2**	ST51-1
			8		1	1		ST21-11
**C15**	**2**	2021	7	**5**	-	-	**1**	ST1328-1
	**3**		3	**14**	3	1	**6**	ST11-2
			4		5	3		ST11-2, ST42-5, ST21-12
			5		3	2		ST19-3, ST21-6
			6		3	1		ST50-3
	**4**		3	**15**	3	1	**5**	ST11-2
			4		5	3		ST11-2, ST10846-1, ST122-1
			5		3	1		ST464-1
			6		4	1		ST50-3
**C16**	**2**	2020	7	3	-	-	**1**	ST1624-1
